# Nontuberculous Mycobacteria in Household Plumbing as Possible Cause of Chronic Rhinosinusitis

**DOI:** 10.3201/eid1810.120164

**Published:** 2012-10

**Authors:** Wellington S. Tichenor, Jennifer Thurlow, Steven McNulty, Barbara A. Brown-Elliott, Richard J. Wallace, Joseph O. Falkinham

**Affiliations:** The Center for Allergy, Asthma and Sinusitis, New York, New York, USA (W.S. Tichenor, J. Thurlow);; New York Medical College, Valhalla, New York (W.S. Tichenor);; University of Texas Health Science Center, Tyler, Texas, USA (S. NcNulty, B.A. Brown-Elliott, R.J. Wallace, Jr.);; Virginia Polytechnic Institute and State University, Blacksburg, Virginia, USA (J.O. Falkinham III)

**Keywords:** chronic rhinosinusitis, nontuberculous mycobacteria, immunodeficiency, household plumbing, CRS, sinusitis, bacteria, *Suggested citation for this article*: Tichenor WS, Thurlow J, McNulty S, Brown-Elliott BA, Wallace RJ, Falkinham JO. Nontuberculous mycobacteria in household plumbing as possible cause of chronic rhinosinusitis. Emerg Infect Dis [Internet]. 2012 Oct [*date cited*]. http://dx.doi.org/10.3201/eid1810.120164

## Abstract

Symptoms of chronic rhinosinusitis (CRS) often persist despite treatment. Because nontuberculous mycobacteria (NTM) are resistant to commonly used antimicrobial drugs and are found in drinking water that patients may use for sinus irrigation, we investigated whether some CRS patients were infected with NTM in New York, New York, USA, during 2001–2011. Two approaches were chosen: 1) records of NTM-infected CRS patients were reviewed to identify common features of infection and *Mycobacterium* species; 2) samples from plumbing in households of 8 NTM-infected patients were cultured for NTM presence. In 3 households sampled, *M. avium* sharing *rep*-PCR and pulsed field gel electrophoresis fingerprints identified *M. avium* isolates clonally related to the patients’ isolates. We conclude that patients with treatment-resistant CRS may be infected with NTM and should have cultures performed for NTM so appropriate therapy can be instituted. In addition, the results suggest that CRS patients can be infected by NTM in their household plumbing.

## Abstract 2

Millions of Americans live with chronic sinus infection. Most infections are caused by either bacteria or fungi. Some of these infections can be hard to treat, eluding medical and surgical treatment and persisting for months or even years. A recent study in New York found that some patients with a chronic sinus infection had tuberculosis-like organisms (mycobacteria) in their sinuses and that the same organisms were also in the tap water at their homes. These mycobacteria can be resistant to commonly used antimicrobial drugs. Doctors should check for mycobacteria in patients with treatment-resistant sinus infection. Patients who flush their sinuses at home should use sterile saline, not tap water.

A subset of patients with chronic rhinosinusitis (CRS) often experience persistent symptoms, despite undergoing many medical and surgical modes of treatment. Current theories regarding the cause of CRS include immunologic reactions to microorganisms (*1*,*2*). Even though they receive various treatments, including antimicrobial drugs and sinus irrigation, many patients continue to be symptomatic (*2*). One possible reason for the persistence of symptoms is the presence of microorganisms that are resistant to typically prescribed antimicrobial drugs, for example, nontuberculous mycobacteria (NTM).

Recovery of NTM from the sinus cavity has been documented in 19 patients, including those with cystic fibrosis (*3*), HIV infection (*4*–*10*), and diabetes (*11*). NTM isolation from the sinus cavity has been rarely reported in immunocompetent, nondiabetic patients who do not have cystic fibrosis (*12*–*15*). One case of infection with NTM is documented in a study by Spring and Miller (*14*). The patient had a 21-year history of rhinosinusitis and exhibited left maxillary facial pain, nasal discharge, and congestion. *Mycobacterium chelonae*, *Staphylococcus aureus*, and *Pseudomonas aeruginosa* were recovered from sinus cultures. Successful treatment ultimately required a 3-year course of multiple intravenous antimicrobial drug combinations and subsequent sinus operations (*14*). Recently, a new member of the *M. abscessus-chelonae* complex, *M. franklinii*, was isolated from patients in the northeastern United States who have chronic sinusitis (*16*).

NTM are environmental opportunistic pathogens found in natural and human-engineered waters, including drinking water distribution systems (*17*) and household plumbing (*18*–*20*). NTM species can be classified into 2 groups on the basis of growth rates; rapidly growing mycobacteria (e.g., *M. chelonae* and *M. abscessus*) form colonies in <7 days at 37°C, and slowly growing mycobacteria (e.g., *M. avium* and *M. intracellulare*) take >7 days at 37°C to form colonies. Because NTM are resistant to commonly used antimicrobial drugs (*21*) and are found in drinking water, they might be responsible for antimicrobial drug–resistant, chronic rhinosinusitis. We report the isolation, identification, and fingerprinting of NTM isolates from patients with CRS and from their household plumbing.

## Methods

### Patient Histories

We reviewed the charts of 33 adult outpatients in whom CRS was diagnosed in the medical practice of W.S. Tichenor, whose endoscopically directed sinus cultures yielded NTM. The 33 represent ≈1% of patient samples collected over a 10-year period. In all patients, CRS had been diagnosed on the basis of a combination of initial evaluation; appearance of the sinuses by endoscopic examination; results of computed tomographic scan; and endoscopically directed cultures for bacteria, fungi, and NTM. From all patients, bacterial isolates had been cultured at the time of endoscopy.

Initial symptoms, NTM identity, surgical history, HIV status, cystic fibrosis history and carrier status, diabetes and immune-deficiency status, current nasal irrigations, presence of polyps, treatment, repeat culture results, and symptom reduction were assessed (Table 1). Common patient conditions at the time of nasal endoscopy included headache, nasal blockage or congestion, thick postnasal drip, and decreased ability to taste or smell. Thirty (91%) of the 33 patients had previously undergone endoscopic sinus surgery; 10 (30%) had histories of primary immunodeficiency. Twelve (36%) of the 33 patients had evidence of polyps at the time of nasal endoscopy; however, no clear association was found between NTM species and the presence of polyps. Thirty-one (94%) of 33 patients were using some form of nasal irrigation at the time of endoscopy. Of those, 26 were known to have used tap water to irrigate the sinuses.

**Table 1:** Characteristics of patients whose sinuses yielded NTM in study of NTM in household plumbing, New York, New York, USA, 2001–2011*

Characteristics	Value
Total patients	33 (100)
Age range, y	25–74
Prior functional endoscopic sinus surgery	30 (91)
Nasal polyps	12 (36)
Primary immunodeficiency	10 (30)
HIV positive	0†
Cystic fibrosis carrier state	1 (20)‡
Diabetes	0
Repeat culture NTM negative	21 (64)
Repeat culture NTM positive	2 (6)
Repeat culture not performed or lost	10 (30)
Symptoms improved	14 (42)
Symptoms unchanged	6 (18)
Other persistent microorganism§	1 (3)
Refused treatment	3 (9)
Currently treated	9 (27)

*Values are no. (%) patients except as indicated. NTM, nontuberculous mycobacteria. †No patients were known to be HIV positive; 16 were tested. ‡No patients were known to have cystic fibrosis; 5 were tested. §Methicillin-resistant *Staphylococcus aureus*.

### Patient Sample Collections

Endoscopically directed samples were taken directly from the sinuses, middle meatus, or ostiomeatal unit by using a flexible catheter with a self-contained Lukens trap as described (*22*). Samples (0.5–3 mL) were sent to the microbiology laboratories (Mayo Medical Laboratories, Rochester, MN, USA; Specialty Laboratories, Valencia, CA, USA; Quest Laboratories, Peterboro, NJ, USA) in sterile 5-mL containers.

### Household Collections

Members of households with occupants who had NTM-associated CRS volunteered to participate in studies of their households’ water systems. Informed consent was obtained from each collaborating patient, and the study was reviewed by the Virginia Tech Institutional Research Board and granted exempt status. NTM isolates from the patients were obtained through laboratories that cultured NTM from endoscopy samples. Containers, swabs, and tubes were sent to each collaborating patient’s household. Directions were provided for the collaborating patient or family member to collect hot and cold water samples (500 mL) and biofilms/sediment from water taps and showerheads. Biofilm samples were collected by swabbing the inside of taps and showerheads, and swab specimens were placed in tubes containing 2 mL of tap water (from Blacksburg, VA, USA), sterilized by autoclaving. If in-line or point-of-use water filters were submitted by the patients, a 4-cm^2^ area was swabbed, and the swab was placed in 2 mL of sterile tap water.

### NTM Isolation, Identification, and Fingerprinting

Patient NTM isolates were identified by various methods, depending on the laboratory: DNA probe, high-performance liquid chromatography, gas-liquid chromatography, internal transcribed spacer region or 16S rDNA sequencing. NTM in water and swab (taps and filters) samples were enumerated and isolated as described (*19*). Household NTM isolates and those from patients were identified by nested PCR of 16S rRNA gene (*23*) and PCR amplification and analysis of restriction endonuclease digestion fragments of the *hsp-65* gene (*24*). When the *Mycobacterium* species of the patient and household water system isolates were identical, isolates were fingerprinted by *rep-*PCR (*25*) and pulsed-field gel electrophoresis (PFGE) of *Ase*I and *Xba*I restriction endonuclease digests of genomic DNA (*26*). To interpret PFGE in categories of “indistinguishable,” “closely related,” and “different,” we used previously described criteria for the evaluation of *Mycobacterium avium* complex isolates (*27*). With a minimum of 10 interpretable bands, strains were interpreted as indistinguishable (no band differences), closely related (1–3 band differences), possibly related (4–6 band differences), and different (>7 band differences). These isolates underwent species confirmation by sequencing of the internal transcribed spacer 1. *M. intracellulare* and *M. chimaera* are indistinguishable without gene/region sequencing (*28*).

## Results

Review of the charts of the 33 CRS patients showed that 39 NTM isolates belonging to 10 *Mycobacterium* species were recovered from samples from the ostiomeatal unit or paranasal sinuses (Table 2). The patients’ mycobacterial isolates were identified by Mayo Clinic, Quest, and Specialty Laboratories. Two different *Mycobacterium* species were isolated from 6 patient samples (Table 2). Most isolates (25 [64%] of 39) were rapidly growing mycobacteria, primarily *M. abscessus* or *M. chelonae*. One laboratory that received patient samples did not distinguish *M. abscessus* from *M. chelonae*. The predominant slowly growing *Mycobacterium* species was MAC (6 [15%] of 39). *M. gordonae* was isolated from 4 (12%) of the 33 patients. Although the organism is normally considered a saprophyte, *M. gordonae* infection has been reported in immunodeficient persons (*29*–*31*), and thus its isolation should not be dismissed.

**Table 2:** NTM isolated from sinus cavity samples of 33 patients in study of NTM in household plumbing, New York, New York, USA, 2001–2011*

NTM species	No. (%) patients
*Mycobacterium abscessus-chelonae*	19 (58)
*M. chelonae*	4 (12)
*M. abscessus*	2 (6)
*M. avium*	4 (12)
*M. avium* complex	2 (6)
*M. immunogenum*	1 (3)
*M. asiaticum*	1 (3)
*M. mucogenicum*	1 (3)
*M. mageritense*	1 (3)
*M. gordonae*	4 (12)

*NTM, nontuberculous mycobacteria.

### NTM Isolates from Households of Current CRS Patients

**Figure 1:**
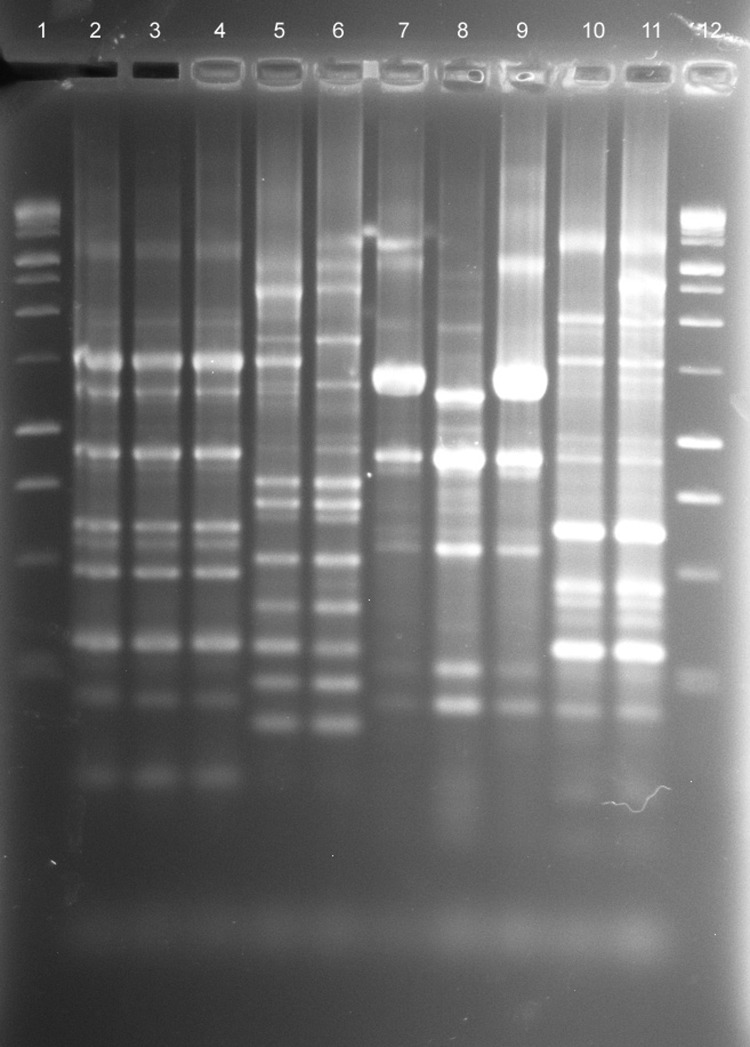
*rep*-PCR fingerprint patterns of patient and household isolates, New York, New York, USA, 2001-2011. Lane 1, 100-bp ladder; lane 2, patient no. 5 *Mycobacterium avium* isolate AG-P-1; lane 3, patient no. 5 household filter *M. avium* isolate AG-F-2–0-2; lane 4, patient no. 5 household filter *M. avium* isolate AG-F-2-I-1; lane 5, patient no. 6 *M. avium* complex “X” cluster (MAC-X) isolate GG-P-1; lane 6, patient no. 6 household swab *M. chimaera* isolate GG-Sw-9–1; lane 7, patient no. 8 *M. avium* isolate GW-P-1; lane 8, patient no. 8 household water *M. avium* isolate GW-W-1–1; lane 9, patient no. 8 household swab *M avium* isolate GW-Sw-7–2; lane 10, patient no. 2 *M. avium* isolate BB-P-1; lane 11, patient no. 2 household water *M. avium* isolate BB-W-4–5; lane 12, 100-bp ladder.

**Figure 2:**
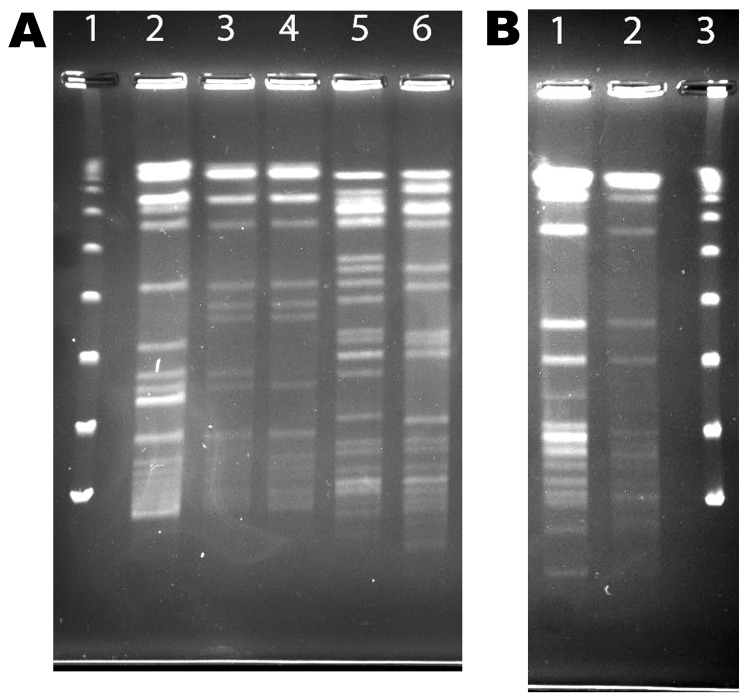
Pulsed-field gel electrophoresis (PFGE) of *Ase*I digest patterns of patient and household isolates, New York, New York, USA, 2001–2011. A) Patient and household isolates. Lane 1, λ ladder; lane 2, patient no. 5 *Mycobacterium avium* isolate AG-P-1; lane 3, patient no. 5 household filter *M. avium* isolate AG-F-2-0-2; lane 4, patient no. 5 household filter *M. avium* isolate AG-F-2-I-1 (environmental isolates in lanes 3 and 4 are indistinguishable; patient isolate in lane 2 considered clonal with 2 environmental isolates [6 bands difference]); with digestion with *XbaI*, the 3 were considered closely related.); lane 5, patient no. 6 *M. avium* complex “X” cluster (MAC-X) isolate GG-P-1; lane 6, patient no. 6 household swab *M. chimaera* isolate GG-Sw-9–1 (despite overall similarity, isolates in lanes 5 and 6 belong to different species and differ by 10 bands and are therefore unrelated). B) Additional patient and isolate from the person’s household. Lane 1, patient no. 2 *M. avium* isolate BB-P-1; lane 2, patient no. 2 household water *M. avium* isolate BB-W-4–5; lane 3, λ ladder.

A total of 80 samples (i.e., 43 water, 31 biofilm, and 6 from filters) for NTM isolation were received from the 8 collaborating CRS patients. NTM were isolated from water, biofilm, or filter samples from at least 1 sample from 5 (63%) of the 8 households sampled and from 35 (40%) of the 80 samples (Table 3). The frequency of NTM recovery from water (44%), biofilm (42%), and filter (50%) samples was not significantly different (p = 0.6065, Kruskal-Wallace test). NTM colony counts varied widely in samples from the different households (Table 4). In 4 households, at least 1 of the samples yielded an NTM isolate that was of the same species and had the same *rep*-PCR fingerprint as that of the patient according to published criteria (*25*) (Figure 1). The band patterns illustrate the large number and wide range of *rep*-PCR bands and illustrate the discrimination provided by *rep*-PCR fingerprinting (*25*). To confirm the relatedness between isolates from patient and household plumbing, PFGE was performed (*26*) for the same isolates (Figure 2). The PFGE band pattern of the isolate from patient 2 and the pattern from the patient’s household (lanes 10 and 11) appear almost identical (“closely related”). The PFGE patterns for 2 isolates from the household of patient 5 were “indistinguishable” and are “closely related” (clonal) to the respective patient isolates and thereby clonal (Figure 2, panel A). Isolates from patient 8 and the patient’s household plumbing (not shown) gave faint signals by PFGE with repeat testing and both restriction enzymes. However, the patterns appeared “indistinguishable”(profile not shown). The lack of clear band patterns for the isolates from patient 8 and his or her household plumbing is likely because of the shared characteristic of resistance to lysis in the agar plugs (*26*). The absence of a match for patient 1 (not shown) might be because the person moved throughout the United States, and some places where the patient lived were not sampled. Samples of showerheads were collected from 6 of the 8 households, and although NTM isolates of the same species as that of the patient (i.e., *M. avium*) were recovered from 2 households, none of the showerhead isolates shared the same fingerprint with isolates from the patient. Notably, the samples from the household plumbing of the patients with *M. gordonae* and *M. immunogenum* isolates did not yield any NTM.

**Table 3:** Recovery of NTM from households in study of NTM in household plumbing, New York, New York, USA, 2001–2011*

Patient household no.	Patient isolate	No. samples collected	No. (%) samples yielding NTM	Species found in patient household†	*rep*-PCR match	PFGE match
1	*M. abscessus*	9	5 (55)	None	NA	NA
2	*M. avium*	9	4 (44)	Yes	Yes	Yes
3	*M. immunogenum*	10	0	NA	NA	NA
4	*M. gordonae*	5	2 (40)	Yes	No	–
5	*M. avium*	10	9 (90)	Yes	Yes	Yes
6	MAC-X†	21	7 (33)	Yes	Yes	No
7	*M. gordonae*	10	0	NA	NA	–
8	*M. avium*	14	8 (57)	Yes	Yes	Yes

*NTM, nontuberculous mycobacteria; NA, not applicable; MAC-X, *Mycobacterium avium* complex “X” cluster; PFGE, pulsed-field gel electrophoresis.  †MAC-X is a mycobacterium that tests positive by DNA probe analysis for *M. avium* complex but is negative with specific *M. avium* and *M. intracellulare* probes and PCR analysis.

**Table 4:** Numbers of NTM in household samples in study of NTM in household plumbing, New York, New York, USA, 2001–2011*

Patient household no.	Water			Biofilm	
	No.	Average CFU/mL		No.	Average CFU/cm^2^
1	4	5,632 ± 3,372		2	36,000 ± 49,500
2	5	49 ± 18			6 ± 2
3	0			0	
4	1			0	
5	13	420 ± 1,000		8	23,310 ± 41,700
6	3	17,052 ± 11,200		11	21,100 ± 27,700
7	0			0	
8	7	27 ± 26		8	513 ± 632
Total	33	2,487		31	13,835

*NTM, nontuberculous mycobacteria.

## Discussion

Our study confirms the possibility of the involvement of NTM in sinuses of patients with CRS (*3*,*11*,*16*). CRS patients who have not responded to medical treatment should undergo endoscopically directed sinus cultures for microorganisms, including fungi and NTM and other bacteria. Endoscopically directed sinus cultures have been shown to accurately replicate sinus puncture culture techniques (*22*). The American Thoracic Society and the Infectious Diseases Society of America discourage the use of swabs for sampling because swabs may decrease the likelihood of recovering NTM (*21*). Using a suction device to remove larger volumes of mucus helps increase the chances of obtaining representative sinus microflora (*22*). Spurious recovery of NTM, because of endoscope contamination, is possible (*32*), as is the possibility that glutaraldehyde may not adequately kill NTM (*33*). However, in the current study, endoscope contamination is an unlikely source of NTM because water samples from the physician’s office did not reveal NTM. In addition, the patient and household samples were processed in different laboratories.

Besides establishing NTM as a potential agent of CRS, our results strongly suggest that in 3 of the 8 CRS patients studied here, the household plumbing was the source of infection, on the basis of identity of *rep*-PCR fingerprints of patient and household isolates and their clonal relatedness as determined by PFGE. Clonal variation in *Mycobacterium* species isolates is characteristic of isolates recovered from household plumbing, but because single *Mycobacterium* species isolates are typically recovered from patient samples, DNA fingerprint matches are not always obtained (*19*,*20*). A study of persons with NTM pulmonary disease found that in 7 (41%) of 17 households, patient and household plumbing isolates were identical as shown by *rep*-PCR fingerprints (*20*). Because NTM are found in household tap water (*19*,*20*,*34*), CRS patients should avoid sinus irrigation with unsterilized tap water.

A major question concerning isolation of NTM from the sinus cavities of patients with CRS is whether NTM were involved in pathogenesis. No guidance exists for the diagnosis and treatment of NTM sinus infection. For pulmonary NTM disease, it is recommended that multiple cultures be obtained over time (*21*) to rule out transient colonization and avoid sampling deficiencies. Our experience suggests that multiple cultures may be necessary to find NTM because endoscopy samples from many patients will be found NTM positive only by 1 of 2–5 endoscopies. For example, 1 patient had cultures that yielded *M. mageritense*, but cultures obtained 1 week later were negative, even in the absence of antibacterial drug treatment. In addition, smears from 2 patients showed acid-fast bacilli, but cultures failed to yield any *Mycobacterium* species isolate; yet upon subsequent endoscopy, NTM were cultured. Several possible reasons could account for this low yield. First, hydrophobic NTM cells are likely adhering to the walls of the sinus cavity, and thereby a low number are in fluid removed during endoscopy. Second, the small volume of mucus removed at the time of culture also might reduce the likelihood of recovering NTM (*22*). Third, topical anesthetics, typically lidocaine, are used for anesthesia for endoscopy and might inhibit the growth of many microorganisms, including NTM (*35*). Although NTM could merely be colonizing the sinuses, several factors suggest otherwise. The samples that we collected were primarily mucus, and previously published reports on NTM in sinus samples from immunocompromised CRS patients were primarily based on biopsy specimens (*3*,*6*,*15*). In addition, our patients typically have persistent symptoms despite treatment with multiple different antimicrobial drug regimens over several months. Because the results of NTM culture and sensitivity testing take several months to obtain, patients are typically treated for other possible infecting microorganisms until the results of the NTM cultures are reported. Resolution typically occurred only after an extended course of multiple antimycobacterial agents given simultaneously. Unfortunately, the combination of insufficient experience and the absence of an established treatment protocol for CRS caused by NTM (*21*), prevent any meaningful review of treatment regimens for CRS caused by NTM. Such patients are treated with 2 oral antimycobacterial drugs and urged to irrigate sinuses with sterile or boiled water or saline, followed by irrigation with a topical antimycobacterial agent for 3–18 months, depending on clinical response and, in some cases, on subsequent positive cultures for NTM

The role of NTM in infectious disease processes is only starting to be recognized. This work documents that a proportion of patients with CRS could be infected with NTM and that sinus samples should be cultured for NTM. In addition, CRS patients should avoid sinus irrigation with tap water because tap water may contain NTM, and it may not be possible to remove it. Sterile saline should be used instead.

## References

[1] Bachert C, Zhang N, Patou J, van Zele T, Gevaert P. Role of staphylococcal superantigens in upper airway disease. Curr Opin Allergy Clin Immunol. 2008;8:34–8. 10.1097/ACI.0b013e3282f4178f 18188015

[2] Ponikau JU, Sherris DA, Kephart GM, Kern EB, Congdon DJ, Adolphson CR, Striking deposition of toxic eosinophil major basic protein in mucus: implications for chronic rhinosinusitis. J Allergy Clin Immunol. 2005;116:362–9. 10.1016/j.jaci.2005.03.049 16083791

[3] Brown SM, DeCelie-Germana JK, Shikowitz MJ, Zahtz GD. Nontuberculous mycobacterial sinusitis in children with cystic fibrosis. Int J Ped Otorhinol Extra. 2007;2:9–13. 10.1016/j.pedex.2006.10.002

[4] Cibrián F, Quiles I, Anaut P, Gainzaráin J, Vega L, Andia A. Sinusitis caused by *Mycobacterium avium–M. intracellulare* in a patient with HIV infection. Enferm Infecc Microbiol Clin. 1996;14:401–2. 8756227

[5] Li C, Szuba M, Schuman P, Crane L, Vazquez JA. *Mycobacterium kansasii* sinusitis in a patient with AIDS. Clin Infect Dis. 1994;19:792–3. 10.1093/clinids/19.4.792 7803654

[6] Naguib MT, Byers JM, Slater LN. Paranasal sinus infection due to atypical mycobacteria in two patients with AIDS. Clin Infect Dis. 1994;19:789–91. 10.1093/clinids/19.4.789 7803653

[7] Sussman SJ. Sinusitis caused by *Mycobacterium avium-intracellulare* in a patient with human immunodeficiency virus. Pediatr Infect Dis J. 1995;14:726–7. 10.1097/00006454-199508000-00024 8532443

[8] Tsi L, Gálvez A, Broto J, García Restoy E, Gual J. Sinusitis in HIV infection. Acta Otorrinolaringol Esp. 1994;45:301–2. 7917486

[9] Upadhyay S, Marks S, Arden R, Crane L, Cohn A. Bacteriology of sinusitis in human immunodeficiency virus–positive patients: implications for management. Laryngoscope. 1995;105:1058–60. 10.1288/00005537-199510000-00009 7564835

[10] Zurlo JJ, Feuerstein IM, Lebovics R, Lane HC. Sinusitis in HIV-1 infection. Am J Med. 1992;93:157–62. 10.1016/0002-9343(92)90045-D 1353944

[11] Ferguson BJ, Kapadia SB, Carrau RL. *Mycobacterium avium* complex infection of the paranasal sinuses. Otolaryngol Head Neck Surg. 1997;117:S160–2. 10.1016/S0194-5998(97)70089-5 9419135

[12] Eron LJ, Huckins C, Park CH, Poretz DM, Gelman HK, Ball MF. *Mycobacterium chelonei* infects the maxillary sinus: a rare case. Va Med. 1981;108:335–8. 7257552

[13] Mra Z, Roach J, Brook A. Infectious and neoplastic diseases of the sphenoid sinus—a report of 10 cases. Rhinology. 2002;40:34–40. 12012952

[14] Spring PM, Miller RH. Initial report of primary sinusitis caused by an atypical pathogen (*Mycobacterium chelonae*) in an immunocompetent adult. Ear Nose Throat J. 1999;78:358–9, 362–4. 10355197

[15] Weiss RL, Zahtz GD, Isenberg H. Nontuberculous mycobacterial infection of the frontal sinus in a child. Otolaryngol Head Neck Surg. 1997;116:110–2. 10.1016/S0194-5998(97)70360-7 9018268

[16] Simmon KE, Brown-Elliott BA, Ridge PG, Durtschi JD, Mann LB, Slechta ES, *Mycobacterium chelonae-abscessus* complex associated with sinopulmonary disease, northeastern USA. Emerg Infect Dis. 2011;17:1692–700. 10.3201/eid1709.101667 21888796 PMC3322061

[17] Falkinham JO III. Surrounded by mycobacteria: nontuberculous mycobacteria in the human environment. J Appl Microbiol. 2009;107:356–67. 10.1111/j.1365-2672.2009.04161.x 19228258

[18] Nishiuchi Y, Maekura R, Kitada S, Tamaru A, Taguri T, Kira Y, The recovery of *Mycobacterium avium-intracellulare* complex (MAC) from the residential bathrooms of patients with pulmonary MAC. Clin Infect Dis. 2007;45:347–51. 10.1086/519383 17599313

[19] Falkinham JO III, Iseman MD, De Haas P, van Soolingen D. *Mycobacterium avium* in a shower linked to pulmonary disease. J Water Health. 2008;6:209–13. 18209283 10.2166/wh.2008.032

[20] Falkinham JO III. Nontuberculous mycobacteria from household plumbing of patients with nontuberculous mycobacterial disease. Emerg Infect Dis. 2011;17:419–24. 10.3201/eid1703.101510 21392432 PMC3166028

[21] Griffith DE, Aksamit T, Brown-Elliott BA, Catanzaro A, Daley C, Gordin F, An official ATS/IDSA statement: diagnosis, treatment, and prevention of nontuberculous mycobacterial diseases. Am J Respir Crit Care Med. 2007;175:367–416. 10.1164/rccm.200604-571ST 17277290

[22] Tichenor WS, Adinoff A, Smart B, Hamilos DL. Nasal and sinus endoscopy for medical management of resistant rhinosinusitis, including postsurgical patients. J Allergy Clin Immunol. 2008;121:917–27. 10.1016/j.jaci.2007.08.065 17981318

[23] Wilton S, Cousins D. Detection and identification of multiple mycobacterial pathogens by DNA amplification in a single tube. PCR Methods Appl. 1992;1:269–73. 1282431 10.1101/gr.1.4.269

[24] Telenti A, Marchesi F, Balz M, Bally F, Böttger EC, Bodmer T. Rapid identification of mycobacteria to the species level by polymerase chain reaction and restriction enzyme analysis. J Clin Microbiol. 1993;31:175–8. 8381805 10.1128/jcm.31.2.175-178.1993 PMC262730

[25] Cangelosi GA, Freeman R, Lewis KN, Livingston-Rosanoff D, Shah KS, Milan SJ, Evaluation of high-throughput repetitive sequence–based PCR system for DNA fingerprinting of *Mycobacterium tuberculosis* and *Mycobacterium avium* complex strains. J Clin Microbiol. 2004;42:2685–93. 10.1128/JCM.42.6.2685-2693.2004 15184453 PMC427895

[26] Mazurek GH, Hartman S, Zhang Y, Brown BA, Hector JSR, Murphy D, Large DNA restriction fragment polymorphism in the *Mycobacterium avium–M. intracellulare* complex: a potential epidemiologic tool. J Clin Microbiol. 1993;31:390–4. 8094398 10.1128/jcm.31.2.390-394.1993 PMC262771

[27] Wallace RJ, Zhang Y, Brown-Elliott BA, Yakrus MA, Wilson RW, Mann L, Repeat positive cultures in *Mycobacterium intracellulare* lung disease after macrolide therapy represent new infections in patients with nodular bronchiectasis. J Infect Dis. 2002;186:266–73. 10.1086/341207 12134265

[28] Tortoli E, Rindi L, Garcia MJ, Chiaradonna P, Dei R, Garzelli C, Proposal to elevate the genetic variant MAC-A, included in the *Mycobacterium avium* complex, to species rank as *Mycobacterium chimaera* sp. nov. Int J Syst Evol Microbiol. 2004;54:1277–85. 10.1099/ijs.0.02777-0 15280303

[29] Weinberger M, Berg SL, Feuerstein IM, Pizzo PA, Witebsky FG. Disseminated infection with *Mycobacterium gordonae*: report of a case and critical review of the literature. Clin Infect Dis. 1992;14:1229–39. 10.1093/clinids/14.6.1229 1623079

[30] Maslo C, Hadacek B, Maresca A, Vallee E, Coulaud JP. Infections à *Mycobacterium gordonae* au cours de l’infection par le virus de l’immunodéficience humaine. Presse Med. 1995;24:1157–60. 7567831

[31] Eckburg PB, Buadu EO, Stark P, Sarinas PSA, Chitkara RK, Kuschner WG. Clinical and chest radiographic findings among persons with sputum culture positive for *Mycobacterium gordonae.* Chest. 2000;117:96–102. 10.1378/chest.117.1.96 10631205

[32] Wallace RJ Jr, Brown B, Griffith D. Nosocomial outbreaks/pseudo-outbreaks caused by nontuberculous mycobacteria. Annu Rev Microbiol. 1998;52:453–90. 10.1146/annurev.micro.52.1.453 9891805

[33] Griffiths PA, Babb JR, Bradley CR, Fraise AP. Glutaraldehyde-resistant *Mycobacterium chelonae* from endoscope washer disinfectors. J Appl Microbiol. 1997;82:519–26. 10.1046/j.1365-2672.1997.00171.x 9190297

[34] Feazel LM, Baumgartner LK, Peterson KL, Frank DN, Harris JK, Pace NR. Opportunistic pathogens enriched in showerhead biofilms. Proc Natl Acad Sci U S A. 2009;106:16393–9. 10.1073/pnas.0908446106 19805310 PMC2752528

[35] Schmidt RM, Rosenkranz HS. Antimicrobial activity of local anesthetics: lidocaine and procaine. J Infect Dis. 1970;121:597–607. 10.1093/infdis/121.6.597 4393033

